# Effectiveness and Clinical Indications of 2 × 4 Fixed Orthodontic Therapy in Regard to Mixed Dentition: A Systematic Review

**DOI:** 10.3390/children12070897

**Published:** 2025-07-07

**Authors:** Gianna Dipalma, Grazia Marinelli, Lucia Casamassima, Paola Nardelli, Danilo Ciccarese, Paolo De Sena, Francesco Inchingolo, Vito Crincoli, Andrea Palermo, Ioana Roxana Bordea, Andrea Carbonara, Angelo Michele Inchingolo, Alessio Danilo Inchingolo

**Affiliations:** 1 Department of Interdisciplinary Medicine, University of Bari “Aldo Moro”, 70124 Bari, Italy; gianna.dipalma@uniba.it (G.D.); graziamarinelli@live.it (G.M.); lucia.casamassima@uniba.it (L.C.); paola.nardelli@uniba.it (P.N.); danilo.ciccarese@uniba.it (D.C.); paolo.desena@uniba.it (P.D.S.); francesco.inchingolo@uniba.it (F.I.); vito.crincoli@uniba.it (V.C.); andrea.carbonara@libero.it (A.C.); a.inchingolo3@studenti.uniba.it (A.M.I.); alessiodanilo.inchingolo@uniba.it (A.D.I.); 2 Department of Experimental Medicine, University of Salento, 73100 Lecce, Italy; andrea.palermo@unisalento.it; 3 Department of Oral Rehabilitation, Faculty of Dentistry, University of Medicine and Pharmacy, 400012 Cluj-Napoca, Romania

**Keywords:** mixed dentition, early mixed dentition, 2 × 4 appliance, fixed appliance, orthodontic brackets, interceptive orthodontic treatment

## Abstract

Background/Objectives: This systematic review examines the effectiveness and clinical indications of the 2 × 4 fixed orthodontic appliance for interceptive treatment in children with mixed dentition, aiming to simplify future orthodontic needs. Methods: Following the PRISMA guidelines, a search was conducted across PubMed, Web of Science, and Scopus (May 2000–May 2025). The inclusion criteria focused on open-access, in vivo/human studies, case–control studies, cohort studies, and RCTs in English. Risk of bias was assessed using Rob 2.0. Results: Out of 362 initial records, 7 studies were included. Most of the included studies showed a low risk of bias, with some moderate risk in terms of confounding variables and participant selection, but no high risk was identified. Conclusions: The 2 × 4 fixed orthodontic appliance is an effective interceptive therapy for mixed dentition. Clinicians can optimize outcomes by understanding its biomechanics and clinical applications. Further research is needed to understand long-term impacts and improve efficiency.

## 1. Introduction

During the mixed dentition (MD) phase, children experience the simultaneous presence of primary and permanent teeth, marking a transitional period that is often associated with the development of malocclusions [1,2,3,4,5,6]. Early identification and management of these conditions are crucial to guiding proper occlusal development and reducing the need for more complex treatments in adolescence or adulthood. Interceptive orthodontic treatment plays a vital role in this phase by positively influencing jaw growth and tooth eruption [7,8,9,10,11].

Because primary and permanent teeth coexist throughout the MD period, there is a special opportunity for early orthodontic intervention [12,13,14,15,16,17,18]. In order to address emerging malocclusions, the craniofacial complex’s growth and development can be altered during this time. Improving general dental and skeletal relationships, lowering the risk of tooth impaction, and lessening the degree of malocclusions can all be achieved with prompt intervention. Because the 2 × 4 appliance may use the stability of the first permanent molars to manage the alignment and position of the anterior teeth, it is very helpful during this phase [19,20,21,22,23].

The biomechanical concepts of the 2 × 4 appliance will be examined in this review, along with how forces are applied to produce the necessary tooth movements. The clinical indications for its use, such as the treatment of mild to moderate crowding, the alignment of ectopic or malpositioned incisors, and the repair of anterior crossbites, will also be thoroughly reviewed. Using current research and clinical data, particular attention will be paid to the appliance’s performance in each of these clinical situations [24,25,26,27,28,29,30,31,32,33]. Additionally, the benefits and drawbacks of the 2 × 4 appliance will be examined, and a comparison to alternative interceptive therapy approaches will be presented. Considering the durability of the obtained outcomes and the influence on future craniofacial growth, the possible long-term implications of the 2 × 4 appliance treatment will also be investigated [34,35,36,37,38].

Additionally, the patient management factors during 2 × 4 appliance installation will be examined in this article, covering methods for reducing discomfort, ways of improving patient compliance, and advice on how to keep one’s teeth clean [39,40,41,42,43,44,45,46,47,48,49]. The effectiveness of any orthodontic treatment, including with the 2 × 4 appliance, depends on a thorough commitment to patient management. Patient participation can be greatly increased, and the best possible treatment results can be guaranteed with clear instructions, effective communication, and continuous support [50,51,52,53,54,55].

The psychological effects of early orthodontic intervention with the 2 × 4 appliance, in addition to its clinical features, will be examined in this research [56,57]. A child’s social relationships and sense of self-worth can be greatly impacted by malocclusions, and treating these problems while children are still in the MD stage can have a major positive impact on their general well-being. By enhancing dental appearance and functionality, the 2 × 4 appliance can help young patients feel more confident and provide them with a more positive self-image [58,59].

**Figure 1:**
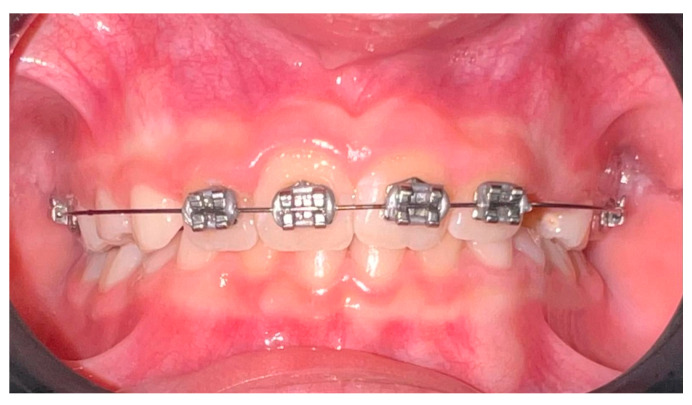
This frontal view of the 2 × 4 fixed orthodontic appliance shows the anterior brackets placed on the upper incisors and the molar bands anchoring the archwire.

**Figure 2:**
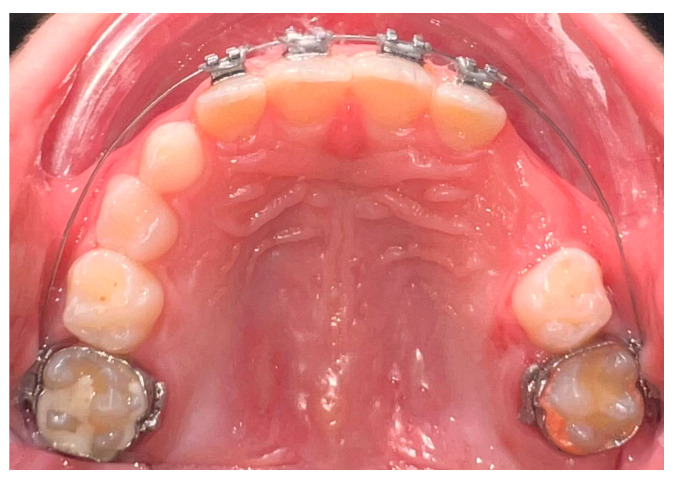
This occlusal view of the 2 × 4 fixed orthodontic appliance illustrates the archwire’s alignment, the brackets’ positions on the incisors, and the molar bands providing posterior anchorage.

The economic factors related to the utilization of the 2 × 4 appliance will also be discussed in this essay. The need for more involved and expensive orthodontic treatment later in life may be lessened by interceptive treatment during the MD phase. The 2 × 4 appliance helps lessen the overall cost burden on patients and their families by treating malocclusions early on (Figure 1 and Figure 2) [60,61,62,63,64,65].

The primary objective of this systematic review is to assess the clinical effectiveness and appropriate indications of the 2 × 4 fixed orthodontic appliance in the interceptive treatment of children during the mixed-dentition phase [66,67,68,69,70,71,72,73]. Our aim is to synthesize current evidence from the literature to clarify when and how this appliance is most beneficial and evaluate the outcomes associated with its use [74,75,76,77,78,79,80].

## 2. Materials and Methods

### 2.1. Protocol and Registration

The current systematic review (SR) was conducted in accordance with the PRISMA guidelines (Preferred Reporting Items for SR and Meta-Analyses) and International Prospective Register of SR Registry procedures (ID PROSPERO: 1059757).

### 2.2. Search Process

The following databases were combed from May 2000 to May 2025 to search for articles published over the last 25 years (Table 1): PubMed, Web of Science (WOS), and Scopus. The search strategy was developed by combining terms relevant to the study’s purpose. In the advanced search strings used in the databases, the following keywords were applied, using Boolean operators to combine terms pertinent to this study’s purpose: (“mixed dentition” [All Fields]) AND (“early mixed dentition” [All Fields])) AND (“2 × 4 appliance” [All Fields])) OR (“fixed appliance” [All Fields])) AND (“orthodontic brackets” [All Fields])) OR (“interceptive orthodontic treatment” [All Fields]).

### 2.3. Inclusion and Exclusion Criteria

The reviewers worked in groups to assess all relevant studies that evaluated or compared the effectiveness and clinical Indications of 2 × 4 fixed orthodontic therapy in the MD phase, following the inclusion criteria below:

- Open-access studies written in English;
- Studies conducted in vivo or on humans;
- Case–control studies, cohort studies, and randomized controlled trials (RCTs);
- Studies involving children aged 6 to 12 years, corresponding to the mixed-dentition phase;
- Studies published in the last 25 years.

Studies that fulfilled at least one of the following exclusion criteria were excluded: reviews, case reports and series, letters to the authors, animal models, studies on adults, and in vitro studies.

### 2.4. PICo Question

The PICo format is a framework used in qualitative research to structure clinical research questions. In this study, the PICo addressed the following question: “In children with MD, how effective is fixed orthodontic therapy using the 2 × 4 technique within the context of interceptive orthodontics, and at which clinical stages is its application most appropriate?”

The PICO question was answered as follows:

P (population): Children in the MD stage.

I (intervention): Fixed orthodontic therapy with the 2 × 4 technique.

Co (context): Interceptive orthodontics, with attention paid to the most appropriate clinical timing for intervention

### 2.5. Data Processing

Four independent reviewers (L.C., D.C., P.D.S, and P.N.) assessed the included studies’ quality using selection criteria, methods of outcome evaluation, and data analysis. This enhanced ‘risk of bias’ tool additionally provides quality standards for selection, performance, detection, reporting, and other biases. All differences were settled through conversation or collaboration with other researchers (G.D., V.C., G.M., A.P., F.I., A.D.I., and A.M.I.). The reviewers screened the records according to the inclusion and exclusion criteria. The 1.202 selected articles were uploaded to “Mendeley Reference Manager Version 2.129.0” for organization and analysis.

## 3. Results

### 3.1. Selected Studies and Their Characteristics 

**Figure 3:**
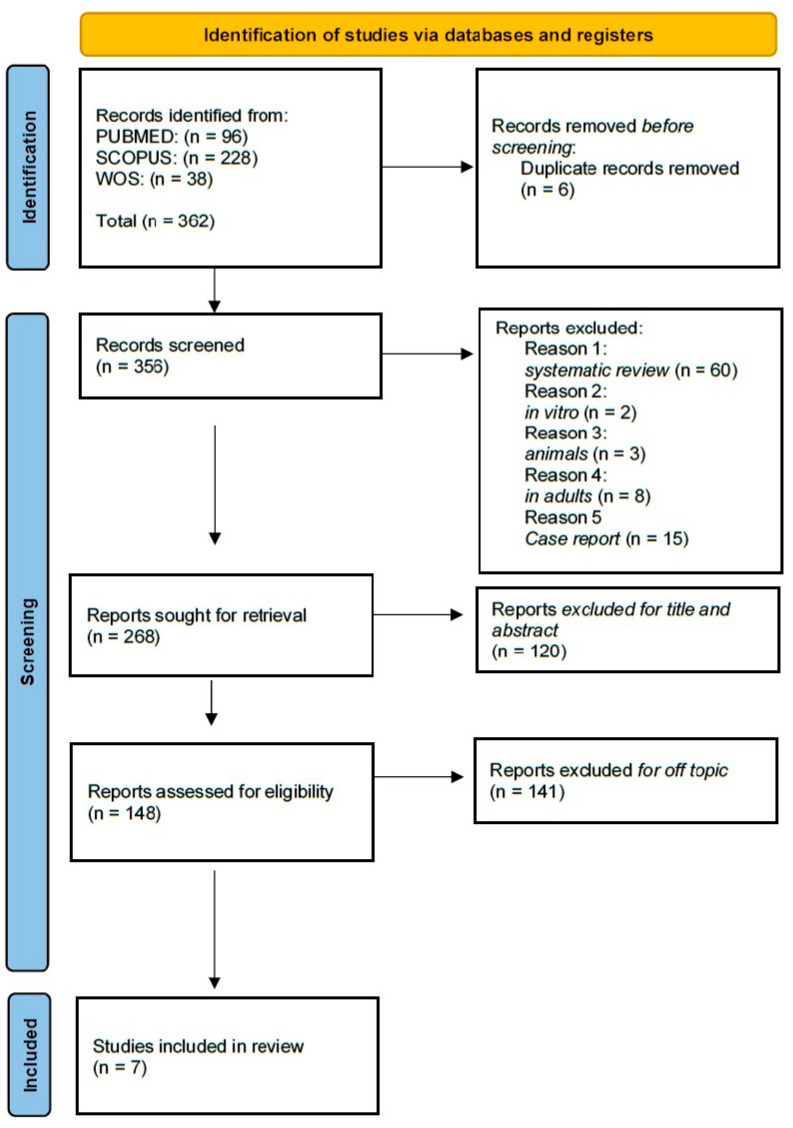
PRISMA flow diagram illustrating the study selection process for the systematic review, including the number of records identified, screened, assessed for eligibility, and included in the final analysis.

This PRISMA (Preferred Reporting Items for SR and Meta-Analyses) diagram (Figure 3) illustrates that a rigorous and systematic selection process was followed to ensure that only relevant studies were included in the final review. A literature search was conducted across three electronic databases: PubMed, Scopus, and Web of Science. This search initially yielded a total of 362 records (96 from PubMed, 228 from Scopus, and 38 from Web of Science). After 6 duplicate entries were removed, 356 unique articles remained for screening. During the initial screening based on titles and abstracts, 88 articles were excluded for various reasons: 60 were SRs, 2 were in vitro studies, 3 involved animal models, 8 focused on adult populations, and 15 were case reports. As a result, 268 articles were considered potentially relevant and thus selected for full-text retrieval. Following this, 120 of these articles were excluded after a more detailed title and abstract evaluation, leaving 148 full-text articles to be assessed for eligibility. Upon thorough review, 140 articles were excluded because they were off-topic or did not align with the inclusion criteria established for this review. Ultimately, seven studies met all the eligibility criteria and were included in the final analysis. The selection process and a summary of the included records are illustrated in Figure 3, while the characteristics of the selected studies are presented in Table 2.

### 3.2. Quality and Risk-of-Bias Assessment for the Included Articles

The quality of the papers included was assessed by a reviewer, P.N., using the Cochrane Risk-of-Bias tool (RoB 2.0), Five points were evaluated, and each was assigned a degree of bias. A senior reviewer (F.I.) was consulted to clear up any doubts. The risk of bias of the included randomized controlled trials pertains to five domains related to the design, conduct, and reporting of clinical trials. Overall, most of the studies demonstrated a low risk of bias across many domains. The studies by Gu et al., 2000 [81], Hägg et al., 2004 [82], and Gu et al., 2005 [83], were considered generally reliable, although some concerns were identified, particularly regarding the measurement of outcomes and the consistency of reporting. Wiedel et al.’s study, 2016 [84], presented some concerns related to the randomization process while maintaining a low risk in the other domains. The studies by Mashouf et al., 2018 [85], and Cruz et al., 2023 [87], demonstrated a low risk of bias in all the areas assessed, indicating high methodological quality. The study by Da Silva et al., 2023 [86], was also considered robust, with low risk in all domains except for outcome measurement, where minor concerns were raised. In summary, the risk of bias was judged to be low in the majority of studies, with only a few domains flagged for minor methodological concerns and no being study classified as having a high overall risk (Table 3).

## 4. Discussion

The reviewed literature, spanning over two decades, reflects a dynamic evolution of our understanding and management of anterior malocclusions during the MD phase. A comparative evaluation of the findings highlights how different appliances and protocols, ranging from fixed 2 × 4 systems to clear aligners and reverse headgear, can be used to achieve similar goals via distinct biomechanical and clinical pathways [88,89,90,91,92,93].

Gu et al., 2000, laid the groundwork in this area by comparing 2 × 4 fixed appliances with reverse headgear among patients with pseudo-class III malocclusion [81]. They found that the 2 × 4 system corrected overjet solely through dental changes, whereas reverse headgear resulted in both skeletal and dental effects (60% and 40%, respectively) [81]. At the one-year follow-up, relapses occurred in the reverse headgear group due to mandibular growth, while the 2 × 4 group maintained stability through dental compensation. Notably, Gu et al., 2000, also reported long-term relapses in reverse headgear cases due to mandibular growth, a dimension largely absent in more recent aligner-focused trials such as that conducted by Da Silva et al., 2023, raising concerns about the lack of longitudinal follow-ups in contemporary research. Gu also developed a predictive cephalometric equation to help determine which patients are more suitable for orthodontic versus orthopedic approaches [81,86,94,95,96,97,98,99,100,101,102,103].

Hägg et al., 2004, confirmed these results with a 5-year follow-up of children treated for pseudo-class III malocclusion using the 2 × 4 appliance. Among the 25 patients who completed the study, 80% required no additional orthodontic treatment [82]. Overjet correction remained stable, and sagittal relationships improved or were maintained. However, greater vertical growth (e.g., increased lower-face height) was observed among patients who required further treatment, suggesting skeletal growth patterns still impact long-term outcomes [82,104,105,106,107,108,109,110]. This nuanced observation suggests that skeletal patterns remain a limiting factor, even in well-controlled orthodontic interventions—an issue less emphasized in studies like those by Cruz et al., 2023, or Mashouf et al., 2018, which tend to report success rates without dissecting the skeletal–dental interplay in detail [85,87].

Mashouf et al., 2018, expanded the evidence base with a retrospective study of 205 children treated during the MD phase using fixed appliances [85]. They reported that 71% of the treatments were completed in a single phase, with only 9% requiring a separate second phase. The extraction rate was exceptionally low (<1%) [111,112,113,114,115,116]. These findings support the use of early interceptive treatment as a cost-effective and broadly applicable strategy for managing malocclusion across classes I, II, and III [85,117,118,119,120,121]. Nevertheless, the absence of detailed skeletal analysis or growth trajectory considerations somewhat limits the clinical specificity of these authors’ conclusions, particularly when compared to the diagnostically driven approach employed by Gu et al., 2005 [83].

Da Silva et al., 2023, introduced a contemporary perspective by comparing the 2 × 4 appliance with clear aligners in a randomized clinical trial [86,122,123,124,125,126,127,128,129]. Both approaches were equally effective in resolving maxillary incisor irregularities, with similar treatment durations (~8 months), arch shape outcomes, and oral hygiene results [130,131,132,133,134,135,136,137,138,139]. No significant overjet increases or adverse occlusal effects were noted in either group, suggesting that clear aligners can serve as a viable alternative for mild to moderate crowding, assuming adequate patient compliance [86,140,141,142,143,144,145,146,147].

Cruz et al., 2023, further enhanced the 2 × 4 method by incorporating NiTi open-coil springs and CuNiTi archwires. Their results demonstrated that this approach led to significant improvements in arch perimeter and depth without causing incisor protrusion. These outcomes support the use of auxiliary mechanics to improve treatment effectiveness, particularly in cases with space deficiencies [87,148,149,150,151,152].

Cruz et al., 2023, used 3D finite element analysis to compare two 2 × 4 techniques: the traditional approach and a modified version with brackets bonded to deciduous molars. The modified technique led to greater anterior tooth movement and superior molar anchorage while also reducing archwire deformation and soft tissue trauma [87,153,154,155,156,157,158,159,160,161,162]. Cruz et al., 2023, extended the 2 × 4 protocol by introducing mechanical innovations, such as NiTi springs and modified anchorage strategies—which indeed improved spatial outcomes. Yet, these studies, while technically informative, provide limited reflection on the long-term stability or biological adaptability of these changes. Wang’s finite element analysis, though biomechanically rigorous, may not fully capture the clinical nuances over time. These findings offer a biomechanical explanation for the improved control seen clinically in Cruz’s study [87,163,164,165,166,167,168].

A clear theme across all studies is the clinical value of early interceptive treatment, especially when using the 2 × 4 appliance. Gu and Hägg established its effectiveness and long-term stability, while Mashouf et al., 2018, demonstrated its efficiency and wide applicability [85]. Da Silva’s comparison with aligners represents a modern shift toward aesthetic and comfort-driven approaches, while Cruz et al., 2023, introduced mechanical innovations to enhance efficiency and control [86,87,169,170,171,172,173,174].

One key divergence lies in the interpretation of skeletal versus dental effects. Gu et al., 2000, placed strong emphasis on distinguishing between the two, while Da Silva and Cruz focused primarily on occlusal and spatial outcomes. Notably, only Gu et al., 2000, provided data on long-term relapses, indicating a gap in more recent studies involving clear aligners [41,81,175,176,177,178,179,180].

Furthermore, only Gu et al. and Wang et al. addressed diagnostic tools and predictive models, indicating future potential for incorporating cephalometric prediction or digital simulations into treatment planning [181,182,183,184,185,186,187].

Wiedel et al., 2016, compared pain, discomfort, and jaw function among children treated for anterior crossbite with fixed or removable appliances. Fixed appliances caused slightly more early pain, especially during eating, while removable appliances had a greater effect on speech and daily activities. Despite these differences, both options were well tolerated, supporting their use based on individual needs and preferences [84,188,189,190,191].

Only Wiedel et al., 2016, shifted the focus away from structural outcomes to patient-centered variables such as pain, comfort, and function [84]. Their balanced view recognizes that treatment success is not just biomechanical but also experiential. Their findings emphasize the need for personalized treatment choices based on tolerance and lifestyle, a point that is mentioned but not fully explored in the other studies [84].

Gu et al., 2005, extended their earlier work by developing a predictive cephalometric model to distinguish which patients with anterior crossbite and mild maxillary deficiency could be successfully treated with a 2 × 4 appliance and which required reverse headgear and maxillary expansion [83]. Through discriminant analysis of cranial base measurements, they formulated a prediction index based on three variables: the cranial base angle (NSAr) and the anterior (S-N) and posterior (S-Ar) cranial base lengths. A positive index score indicated successful treatment with a 2 × 4 appliance, while a negative score suggested the need for orthopedic intervention. The predictive accuracy reached 86.7% for the orthodontic group and 93.3% for the orthopedic group. This quantitative tool not only validated the functional differences between orthodontic and orthopedic correction strategies but also introduced a clinically applicable method for individualized treatment planning, reaffirming the importance of diagnostic precision in early interceptive therapy [49,83,192,193].

In summary, while all the studies agree on the effectiveness of the 2 × 4 appliance for early treatment, their approaches to defining success diverge sharply. Gu’s and Hägg’s emphasis on skeletal growth, relapse, and diagnosis stands in contrast to the procedural efficiency highlighted by Mashouf or the aesthetic equivalence argued for by Da Silva. Likewise, the technological optimism of Cruz and Wang invites innovation but perhaps at the cost of a long-term perspective.

## 5. Conclusions

Our analysis of the current literature and clinical data provides several key insights regarding the use of the 2 × 4 fixed appliance in interceptive orthodontic treatment during the mixed-dentition phase.

The 2 × 4 fixed orthodontic appliance demonstrates strong clinical effectiveness as an interceptive treatment during the mixed-dentition phase, particularly for managing anterior malocclusion [194,195,196,197].

Across various studies, the appliance has shown stable outcomes, a reduced need for extractions, and a high success rate in regard to avoiding second-phase treatments.

While primarily allowing dental corrections, the 2 × 4 appliance can also support skeletal development, depending on the treatment context.

Modern alternatives such as clear aligners show similar clinical efficacy in mild cases and offer aesthetic and comfort advantages, though they require high patient compliance [27,198,199,200,201].

Recent innovations, including the use of auxiliary mechanics and improved materials, have enhanced the biomechanical performance of the 2 × 4 system.

Despite positive short- to medium-term results, long-term data, particularly on relapse rates and skeletal versus dental outcomes, remain limited, underlining the need for continued longitudinal research.

Incorporating diagnostic tools and predictive models into early treatment planning could further personalize care and optimize outcomes.

## 6. Limitations of This Review

As with many systematic reviews, the present study is not without its limitations. One notable concern is the heterogeneity observed among the included studies. Differences in study design, sample size, the age ranges of participants, outcome measures, follow-up duration, and treatment protocols limited our ability to perform a quantitative synthesis or meta-analysis. Additionally, variations in how outcomes were defined and reported—particularly in distinguishing skeletal from dental effects—complicate the comparison and interpretation of the results. While we aimed to apply strict inclusion criteria and conduct a robust methodological assessment, the diversity in the nature and scope of the studies introduced an element of variability that must be considered when interpreting the overall findings.

## Figures and Tables

**Table 1:** Indicators for database searches.

Article-screening strategy	Keywords: “Mixed Dentition; Early Mixed Dentition; 2 × 4 Appliance; Fixed Appliance; Orthodontic Brackets; Interceptive Orthodontic Treatment.”
	Boolean Indicators: OR and AND
	Timespan: May 2000 to May 2025
	Electronic databases: PubMed; Scopus; WOS.

**Table 2:** Summary of the studies selected and included in the systematic review, indicating authors, publication year, study design, sample characteristics, interventions, and main outcomes.

Authors and Years	Type of Study	Patients	Aim of the Study	Materials and Methods	Conclusions
Gu, Y. et al., 2000 [81]	Comparative clinical study	37 children (17 with fixed appliance, 20 with reverse headgear)	Compare overjet correction achieved when using 2 × 4 fixed appliance vs. that achieved using reverse headgear.	Cephalometric analysis at 3 time points and modified Pancherz method.	Both led to similar degrees of overjet correction; fixed appliance changes were dental, and reverse headgear included skeletal changes.
Hägg et al., 2004 [82]	Prospective longitudinal follow-up study	27 children initially (25 completed follow-up); age at start: ~10.1 years; follow-up at ~16.5 years	To assess 5-year outcomes of early pseudo-class III treatment with a 2 × 4 appliance.	A total of 27 patients underwent 2 × 4 treatment, evaluating cephalometrics at T0, T1, and T2. Five needed additional treatment for crowding.	2 × 4 appliance corrected overjet, with stable 5-year results; 25% of patients needed further treatment
Gu et al., 2005 [83]	Clinical comparative study.	30 patients; mean age: ~8.9 years (range 7.1–10.3)	To classify mild maxillary deficiency cases for 2 × 4 appliance or reverse headgear with expansion.	A total of 30 patients with anterior crossbite were treated using either a 2 × 4 appliance or reverse headgear. Analysis led to a predictive equation.	A prediction equation can be used to guide treatment: a positive score suggests the need for a 2 × 4 appliance; a negative score indicates the need for reverse headgear with expansion.
Wiedel, AP. et al, 2016 [84]	Randomized controlled	62 children with anterior crossbite	Compare self-perceived pain and jaw function in relation to fixed vs. removable appliances	Questionnaires conducted over 8 weeks, VAS scales, functional questions, and randomized appliance allocation	Minor differences in discomfort were noted; both appliances were well tolerated and effective.
Mashouf et al., 2018 [85]	Retrospective study	205 patients, aged 6–10 years (mean 8.6 ± 0.7 years)	To assess the outcomes and timing of mixed-dentition orthodontic treatment	Fixing appliance protocol addressing skeletal and dental components; analyzing treatment phases, age, extraction rate, treatment duration, and costs	Early treatment reduced tooth extraction to <1%; was effective across calss I, II, and III; and negated the need for second-phase treatment in 71% of cases. Starting treatment before age 8 improved outcomes
Da Silva, V. et al., 2023 [86]	Randomized clinical trial	32 children (7–11 years old)	Compare efficacy and efficiency of clear aligners vs. 2 × 4 appliances in regard to mixed dentition	Digital dental models and 3D software were used. Patients were randomized into clear aligner or 2 × 4 fixed-appliance groups and treated for ~8 months.	Both methods showed similar efficacy; appliance choice can be based on clinician and family preference.
Cruz, J. et al., 2023 [87]	Prospective non-randomized clinical study	48 children (24 treated, 24 controls)	Evaluate upper-arch dimensional changes using 2 × 4 appliance with NiTi spring in MD phase.	Lateral radiographs, cast models, CuNiTi wires, and NiTi open springs were used. Treated patients were compared to growth-only controls.	2 × 4 appliance effectively increased arch perimeter and depth and maintained incisor position.

**Table 3:** A tabular summary of the risk-of-bias assessment for the 7 studies, evaluated across the five domains of Rob 2.0.

Authors and Year	D1	D2	D3	D4	D5	Overall	
Gu, Y. et al., 2000 [81]	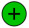	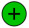	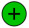	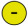	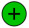	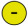	
Hägg et al., 2004 [82]	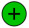	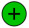	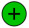	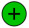	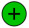	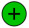	
Gu et al., 2005 [83]	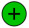	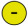	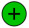	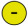	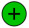	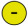	
Wiedel, AP. et al., 2016 [84]	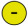	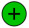	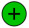	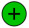	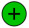	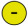	
Mashouf et al., 2018 [85]	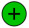	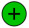	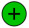	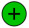	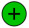	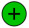	
Da Silva, V. et al., 2023 [86]	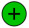	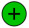	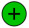	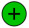	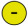	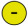	
Cruz, J. et al., 2023 [87]	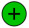	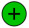	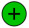	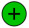	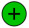	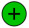	
**Domains:**						**Judgement:**	
D1: Bias arising from the randomization process						Very High	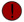
D2: Bias due to deviations from intended interventions						High	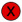
D3: Bias due to missing outcome data						Some Concerns	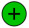
D4: Bias due to missing data.						Low	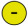
D5: Bias arising from the measurement of the outcome.						No Information	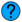

## Data Availability

The data is contained within the article.

## Abbreviations

The following abbreviations are used in this manuscript:

Abbreviation	Definition
MD	Mixed dentition
SR	Systematic review
